# Investigation of the Prevalence, Virulence Genes, and Antibiogram of Motile Aeromonads Isolated from Nile Tilapia Fish Farms in Egypt and Assessment of their Water Quality

**DOI:** 10.3390/ani10081432

**Published:** 2020-08-16

**Authors:** Fatma A. El-Gohary, Eman Zahran, Eman A. Abd El-Gawad, Adel H. El-Gohary, Fatma M. Abdelhamid, Amany El-Mleeh, Ehab Kotb Elmahallawy, Mona Mohieldin Elsayed

**Affiliations:** 1 Department of Hygiene and Zoonoses, Faculty of Veterinary Medicine, Mansoura University, Mansoura 35516, Egypt; adelelgohary@yahoo.com (A.H.E.-G.); dr.monamohy@yahoo.com (M.M.E.); 2 Department of Internal medicine, Infectious and Fish diseases, Faculty of Veterinary Medicine, Mansoura University, Mansoura 35516, Egypt; emanzahran@mans.edu.eg; 3 Aquatic animals diseases and management department, Faculty of Veterinary Medicine, Benha University, Benha 13736, Egypt; dreman_2010@yahoo.com; 4 Department of Clinical Pathology, Faculty of Veterinary Medicine, Mansoura University, Mansoura 35516, Egypt; fatmamostafa980@yahoo.com; 5 Department of Pharmacology, Faculty of Veterinary Medicine, Menoufia University, Sheibin Elkom 32511, Egypt; amany.ahmed1074@gmail.com; 6 Department of Biomedical Sciences, University of León, 24071 León, Spain; 7 Department of Zoonotic diseases, Faculty of Veterinary Medicine, Sohag University, Sohag 82524, Egypt

**Keywords:** *Aeromonas*, Egypt, fish farms, physicochemical water parameters, virulence genes

## Abstract

**Simple Summary:**

Motile *Aeromonas* Septicemia (MAS) has been considered one of the most important bacterial diseases affecting the aquaculture industry in Egypt, and it is also a public health concern. The present study investigated the prevalence, virulence genes, and antibiogram of motile aeromonads isolated from Nile tilapia fish farms in Egypt through bacteriological, molecular, and histopathological identification. The work also involved assessment of the water quality of the examined fish farms. Our results reported *Aeromonas* isolates in 33.3% and 12.5% of fish and water samples, respectively, followed by their molecular identification at the genus level. The recovered motile aeromonads harbored four virulence genes: aerolysin, elastase, hemolysin, and lipase. The antibiogram profile revealed the highest resistance of aeromonads to chloramphenicol, kanamycin, and azithromycin, while lower resistance was recorded against streptomycin, cefotaxime, and amoxicillin. Likewise, severe histopathological changes were evident in liver and spleen that cohere with MAS. Taken together, our data provide interesting information in relation to the adverse impact of water quality and motile aeromonads as a repository of antimicrobial resistance and virulence genes in the aquaculture industry in Egypt.

**Abstract:**

The aquaculture industry is a fast-growing sector in Egypt; however, the progress of this industry is impeded by many challenges such as poor water quality and associated bacterial infections. Among others, Motile *Aeromonas* Septicemia (MAS), caused by aeromonads, is among the most important bacterial diseases affecting aquaculture due to its zoonotic potential. In the present work, motile aeromonads were isolated from water samples (*n*= 8) and Nile tilapia (*n*= 240) in four fish farms (farms I, II, III, and IV) in Kafr El-Sheikh province during the period March to August 2017. This step was followed by investigation of the prevalence and phenotypic, molecular, and histopathological characterization of aeromonads. In addition, antimicrobial susceptibility and virulence gene detection were analyzed. Interestingly, physicochemical water analysis revealed different ranges in relation to the fish farms and seasons. More importantly, *Aeromonas* isolates were phenotypically identified in 33.3% and 12.5% from fish and water samples, respectively. The highest prevalence of motile aeromonads (46.7%) was recorded from farm IV, and only 12.5% of water samples were positive for them. Out of 80 isolates, 65 (81.25%) were molecularly identified at the genus level using gyrase B (*gyr*B). The prevalence of the virulence genes detected in the isolated motile aeromonads was aerolysin (*aer*), 52.2%; elastase (*ahp*), 26.25%; hemolysin (*hyl*), 35%; and lipase (*lip*), 3.75%. The antibiogram profile revealed that the highest resistance of aeromonads isolates (80%) was recorded to chloramphenicol, kanamycin, and azithromycin. Meanwhile, lower resistance levels of 40%, 30%, and 20% were found for streptomycin, cefotaxime, and amoxicillin, respectively. The multiple antibiotic resistance (MAR) index values ranged between 0.27 and 0.82 of motile aeromonads isolates. Furthermore, the histopathological examinations of naturally diseased tilapia revealed widespread hepatocellular necrosis with diffuse, numerous rod-shaped bacteria in liver with melanomacrophages and lymphocytic depletion with edema and hemosiderosis in the spleen. Our findings provide an updated epidemiological baseline for future reference and highlight the likely role of the adverse impact of water quality in the outbreaks of motile aeromonads with special reference to virulence genes and antibiotic resistant traits.

## 1. Introduction

The aquaculture industry is a fast-growing sector, which supplies nearly one-third of the world’s seafood requirements [1]. It also helps address unemployment problems by providing employment opportunities for the growing population [2]. Furthermore, fish constitute a significant part of protein consumption in many places all over the world. At the regional level, Egypt is considered to have one of the largest aquaculture industries in Africa, whereas this industry has grown by 45% over the past few years [3]. Therefore, it is not surprising to mention that there is an increasing concern regarding this industry in Egypt, particularly in relation to the ambitious plans of the Egyptian government to set up new aquaculture farms such as the Birkat Ghalioun fish pond project [4]. This fish pond project is considered the largest farm in the Middle East and is considered one of such plans to meet the increased population demand [4]. Among freshwater species, Nile tilapia is the most common commercially farmed fish in Egypt, supplying over 67% of total fish production [5]. This is mainly owing to their rapid growth rate, high nutritive values, and disease tolerance, promoting higher production levels [5]. With the expansion of aquaculture and due to limited water resources, fish farm intensification has been adopted along with wastewater use such as agriculture drainage water [6]. It should be stressed that wastewater effluents contain different types of xenobiotics that eventually influence the fish health and render them more susceptible to infections with huge losses [6]. Motile aeromonads are ubiquitous bacteria in the aquaculture habitat; however, under stress conditions, they might cause an important hemorrhagic septicemic disease in fish besides being associated with serious public health concerns in humans, as they cause gastroenteritis [7]. It is noteworthy to mention that molecular identification using PCR has been used for the determination of etiologic agents in several diseases, and such methods are able to identify possible genes that encode virulence factors responsible for microbial pathogenesis [8]. Sequencing the housekeeping genes such as the gyrase B (*gyrB)* in aeromonads was documented as a reliable method for identification since the sequencing of 16S rRNA has limited resolution for closely related species [9]. Virulence factors, especially those related to extracellular products, have a crucial role in the translocation of *Aeromonas* spp. in the epithelium, and therefore, they are broadly associated with pathogenicity [10]. Two aeromonads were identified as *A. hydrophila* and *A. veronii* biovar *sobria* using the nucleotide sequence of the *gyrB* gene along with the detection of Lipase (*Lip),* Aerolysin *(aer),* Serine protease *(Ser),* Cytotoxic enterotoxin *(ACT),* and temperature-sensitive protease *(CAI)* virulence genes [11]. In another investigation, *aer* and hemolysin (*hyl*) virulence factors were detected from aeromonads isolates [12]. Taken into account, antimicrobials have been hugely used over the last decades for the prevention and control of bacterial diseases in aquaculture [13], which in turns led to the emergence of antimicrobial-resistant bacteria [14]. Given the above information, the present study was undertaken to investigate the summer prevalence of motile aeromonads in different fish farms receiving poor quality water, at Kafr-El-Sheikh province, Egypt, using phenotypic and molecular characterization together with investigation of the antibiotic resistance patterns and water quality analysis of these farms.

## 2. Materials and Methods

### 2.1. Ethical Considerations

The study protocol was reviewed and approved by the local guidance of the Research Ethics Committee of the Faculty of Veterinary Medicine, Mansoura University, Egypt.

### 2.2. Study Area and Data Collection

The present study was conducted between March and August 2017. A total number of four Nile tilapia fish farms (I, II, III, and IV) located at Kafr-Elsheikh governorate, Egypt were selected, as 55% of the cultured fish production in Egypt is recorded from this area [15]. Fish farms were selected based on the type of fish production, water source, nearby sewage areas, chemotherapy used, and the previous history of fish mortalities in the last two summer seasons. Farm visits were done monthly to cover the whole study period. Fish farmers were interviewed and agreed orally to share in the structured questionnaire, including individual, location, and farm information (Table 1).

### 2.3. Sampling

Nile tilapia (*Oreochromis niloticus*) fish samples were collected monthly throughout the study period (March–August 2017). Two water samples were collected from each farm for the screening of water parameters, one in March and the other in August. About 100 mL of water sample was collected in sterile 500 mL glass bottles, between 10:00 and 12:00 at a depth of 30 cm below the water surface. Each sample was labeled with the following information: number, site, date and time of collection, name of the farm owner, and type of fish production. Then, they were transported to the laboratory in an icebox and stored at 4 °C according to methods mentioned elsewhere [16]. The samples were analyzed within the next 1–2 h or kept in the refrigerator not more than 24 h. Upon farm visits, two ponds were randomly selected; then, five fish were collected per each pond with a total of 60 fish for each farm during the whole study period. Healthy and infected fish were collected with a preference for fish showing signs of Motile *Aeromonas* Septicemia (MAS); then, they were transported alive in a large plastic water container provided with battery aerators as an air source to the laboratory for further investigation. Average lengths and body weights were recorded; then, the clinical examination of fish was performed [17].

### 2.4. Assessment of Water Quality Parameters

The temperature, pH, and dissolved oxygen of water were determined in situ at the farm using a thermometer, pH meter, and oxygen meter (Lovibond^®^, Dortmund, Germany). Other chemical parameters such as total dissolved solids (TDS), conductivity, nitrate, nitrite, ammonia–nitrogen, and phosphorus were measured using a multiparameter bench photometer (HANNA^®^, HI83200-02, Schaumburg, IL, USA).

### 2.5. Bacteriological Examination

A total number of 240 fish samples at a rate of five fish/farm/month and eight water samples were collected during the study period. For bacteriological isolation from water samples, 100 mL was serially diluted; then, 1 mL of the sample was directly streaked on Oxoid^TM^ *Aeromonas* Medium Base (Ryan) with Ampicillin supplement. For the detection of *Aeromonas* from collected tilapia, liver and kidney were sampled and investigated as previously described elsewhere [18]. All collected samples were taken under complete aseptic conditions and seeded directly onto Oxoid^TM^ *Aeromonas* Medium Base (Ryan) with ampicillin supplement 2.5 mg/500 mL of diluted media; then, they were incubated at 28 °C for 24–48 h for the isolation of *Aeromonas* from fish and water. Four to five small dark green convex bull eye-shaped colonies with a dark green center were picked up, purified, and biochemically identified according to the criteria described in Bergy’s Manual of Determinative Bacteriology [19]. Further molecular characterization was carried out on purified biochemically identified strains kept in BHI (brain heart infusion broth) with 15% glycerol at −20 °C [12].

### 2.6. Molecular Identification of the Recovered Aeromonas Isolates

The genomic DNA of *Aeromonas* isolates was extracted using a Gene jet genomic DNA purification kit (Thermo Fisher Scientific Inc., USA) according to the manufacturer’s instructions. The extracted DNA was subjected to *gyrB* gene sequencing for the identification of *Aeromonas* isolates at the genus level, as mentioned elsewhere [20]. *Aeromonas* isolates were recognized for the presence of four virulence genes: *aer*, *hyl*, *lip*, and elastase (*ahp)* using specific primers as described elsewhere [20,21,22]. Aliquots from amplification reactions were analyzed by 1% agarose gel electrophoresis and viewed under UV light. The primer sets, PCR conditions, and size of the amplified sequence are shown in Table 2.

### 2.7. Screening of Antimicrobial Susceptibility of Aeromonas and MAR Index

Twenty representative *Aeromonas* fish isolates (*n* = 5/farm) that showed positive results for *Aeromonas* virulence genes (*aer, hyl*, and *ahp*) were tested for their antimicrobial susceptibility using a Kirby–Bauer disk diffusion method against 11 commercially available antimicrobials on Mueller–Hinton agar (Oxoid, Hampshire, UK) according to the Clinical and Laboratory Standards Institute (CLSI) guidelines [23]. These 11 antibiotic discs (Oxoid, Hampshire, United Kingdom) were chloramphenicol (C; 30 µg), ciprofloxacin (CIP; 5 µg), tetracycline (TE; 5 µg), amoxicillin (AM; 30 µg), cefotaxime (CXT; 30 µg); kanamycin (K; 30 µg); trimethoprim/ sulphamethoxazole (SXT; 25 µg); gentamycin (CN; 10 µg), azithromycin (AZM; 15 µg), streptomycin (S; 10 µg), and imipenem (IPM; 10 µg). Then, the results were evaluated as susceptible (S), intermediate (I), or resistant (R) by measuring the inhibition diameter zone following the guidelines of [23]. The MAR index was calculated by dividing the total number of resistances to antimicrobials by each isolate on the total numbers of tested antimicrobials [24]. The isolates that exhibited resistance to three or more classes of antimicrobials were considered as multidrug-resistant strains [25].

### 2.8. Histopathological Examination of Naturally Diseased Nile Tilapia

Tissue specimens from the liver and spleen of naturally diseased Nile tilapia were excised and fixed in 10% neutral buffered formalin for 24 h. The fixed tissues were then processed for staining with hematoxylin and eosin (H&E) [26]. The tissue sections were examined with a light microscope (Olympus CX41, Olympus Medical systems India Private Limited, Haryana, India) and photographed.

**Table 2:** The primer sets, PCR conditions, and size of the amplified sequence of Aeromonas isolates.

Genes	Primers Sequence ((5′-3′)	PCR Conditions	Product Sizes/(Bp)	Reference
Gyrase B (*gyrB*)	TCCGGCGGTCTGCACGGCGT TTGTCCGGGTTGTACTCGTC	• Initial denaturation 94 °C/5 min. • 30 cycles of denaturation at 94 °C/30 s • Annealing at 59 °C/30 s • Extension at 72 °C/1 min	1100	[20]
Aerolysin (*aer*)	AACCGAACTCTCCAT CGCCTTGTCCTTGTA	• Initial denaturation step at 94 °C/5 min • 30 cycles with denaturation at 94 °C/30 s • Annealing at 54 °C/30 s • Extension at 72 °C/1 min	301	[21]
Elastase (*ahp*)	ACACGGTCAAGGAGATCAAC CGCTGGTGTTGGCCAGCAGG	• Initial denaturation: 94 °C/4 min • 35 cycles of denaturation at 94 °C for 30 s • Annealing at 55.5 °C for 30 s • Extension step at 72 °C for 30 s	540	[27]
Hemolysin (*hyl*)	CACAGCCAATATGTCGGTGAAG GTCACCTTCTCGCTCAGGC	• Same amplification conditions for elastase gene except for annealing at 60.6 °C for 30 s	326	[22]
Lipase (*lip*)	ATCTTCTCCGACTGGTTCGG CCGTGCCAGGACTGGGTCTT	• Same amplification conditions for elastase gene except for annealing at 58.2 °C for 30 s	383–389	[27]

### 2.9. Statistical Analysis

Data were analyzed using simple descriptive statistics such as frequency distribution and percentages. Prevalence of infection was calculated by using the following formula:

Prevalence of infection (%) = No. of infected fish/Total no. of examined fish(1)

## 3. Results

### 3.1. Characteristics of Fish Farms

Farm owners willingly responded to the structured questionnaire in Table 1. This questionnaire aimed to assess the farm status and reveal the relationship between farm characteristics and the incidence rate of *Aeromonas* at the genus level. The results showed that all farm owners were males of middle-age with moderate to high education levels. Upon study, all farms were of semi-intensive production type with a size ranging between 8400 and 14,700 m^2^ and with a stocking density between 10,000 and 18,500 fish/pond. Agriculture drainage runoff was the main water source for all fish farms, but farm IV has sewage source plus agriculture drainage. As a fish management protocol, all farms were dependent on feed supplements with water exchange, but only farm III had water paddles in addition to this management protocol. The average fish production ranged between 3 and 5 tonnes/ha with a final marketing weight that fluctuated between 200 and 375 g, with an average of 289.4 g in the examined farms. All farmers used poultry manure without treatment in fish pond fertilization. The mortality rate in the previous two years was estimated by fish owners as between 200 and 500 fish/day in each pond during summer months, where the highest mortalities (500 fish) were recorded in farm IV. Meanwhile, the mortalities in the year while the study was carried out decreased to a range of 150–350 fish/day. Prophylactic and therapeutic treatments differed among the examined farms, where farm I used humic acid, CaOH powder, probiotics, and feed additives, while farm II used calcium carbonate and manganese. Antibiotics were used in farm III, which used tetracyclines in addition to probiotics, and farm IV used chloramphenicol with humic acid and probiotics. Regarding disinfectants, quaternary ammonium compounds were the most used disinfectants in all the examined farms, but farm IV used calcium hypochlorite. The owner of farm IV stated that he had veterinary supervision lately because of the high mortalities occurring in the last two years. Whereas farm I had an occasional veterinary inspection in case of outbreaks, the owners of farms II and III declared a complete absence of veterinary inspection as they depend on their own experiences and neighbors’ advice from fish farmers.

### 3.2. Water Quality Parameters

Some physicochemical water quality parameters were measured in March and August 2017 in the examined fish farms, which are detailed in Table 3. All water physicochemical parameters were notably higher in August than March. At the farm level in relation to the studying period, Farm III displayed the highest levels of temperature, pH, dissolved oxygen (DO), ammonia, nitrate, and nitrite among all examined farms in August as follows: 30.8 °C, 8.9, 4.7 mg/L, 1.8 mg/L, 0.5 mg/L, and 0.69 mg/L, respectively. Meanwhile, electrical conductivity (EC), TDS, and phosphorus achieved the maximum values 3968 mho/cm, 1978.6 mg/L, and 1.9 mg/L, respectively in farm IV during August.

### 3.3. Fish Examination

The selection of the study period was based mainly on the previous history of outbreaks and mortalities that were encountered during the summer. A total number of 60 fish samples were collected from each farm throughout the study period with slight differences between healthy and infected fish numbers from each farm, depending on the availability of infected fish on each visit. The ranges of lengths and weights were 7.85–16.71 cm and 16.35–196.85 g, respectively (Table S1). Alongside farm visits, fish mortalities were noticed at the water surface and on the banks of fish ponds, where farmers just picked up dead fish and threw them on the banks (Figure S1). A variety of clinical signs were observed on dead fish upon examination, including hemorrhages on the skin and fins, vent opening, detached scales with deep skin ulceration, fin erosions, distended abdomen, and exophthalmia (Figure S2).

### 3.4. Bacteriological and Phenotypic Characterization

Upon bacteriological examination, typical *Aeromonas* colonies were picked up and biochemically examined. The obtained bacterial isolates were Gram-negative, motile bacilli, oxidase, catalase, and aesculin hydrolysis-positive with the ability to ferment glucose with acid and gas production. Biochemical results revealed that *Aeromonas* spp. was recovered from only one water sample (12.5%) and 80 fish samples (33.3%) among the examined samples (Table S2).

### 3.5. Total and Monthly Prevalence of Aeromonas

The total prevalence of motile aeromonads recovered from fish samples was 46.7%, 33.3%, 28.3%, and 25% from farms IV, III, II, and I, respectively (Table 4); whereas the prevalence of motile aeromonads in farm IV was the highest in August, July, and June, (80%, 60%, and 40%, respectively). Meanwhile, only one water sample (12.5%) was positive for motile aeromonads in farm IV (Table 5).

### 3.6. Molecular Identification of Aeromonads

Molecular identification was carried out for 80 fish isolates (*n* = 20/farm). PCR amplification of *gyrB* showed that 65 isolates (81.25%) were positively amplified at 1100 bp (Figure S3). Virulence genes detection revealed that out of the 80 isolates, 42, 21, and 28 motile aeromonads (52.2%, 26.25%, and 35%) harbored *aer, ahp,* and *hyl* genes, respectively. Meanwhile, *lip* gene positive isolates were lower and reached only three positive isolates (3.75%) (Table 6).

### 3.7. Antibiotic Resistance Patterns of Motile Aeromonads

The antibiogram profile of the 20-representative typical *Aeromonas* isolates (5/farm) against 11 antimicrobials is presented in Table 7. Our findings revealed that *Aeromonas* spp. were highly resistant (80%) to chloramphenicol, kanamycin, and azithromycin, followed by 75% of the isolates exhibiting resistance to trimethoprim–sulphamethoxazole. About 70% of *Aeromonas* isolates showed resistance to both ciprofloxacin and gentamycin, and 65% displayed resistance to tetracycline. Approximately, 70% of *Aeromonas* isolates had resistance to ciprofloxacin, and 65% were tolerant to both gentamycin and tetracycline. Lower resistance levels of 40%, 30%, and 20% were observed among examined isolates to streptomycin, cefotaxime, and amoxicillin, respectively. However, only two isolates (10%) were resistant to imipenem. According to the obtained results of antimicrobial testing, a multiple antimicrobial resistance (MAR) index was determined. Table 7 showed that the multidrug resistance pattern of *Aeromonas* isolates and MAR index values ranged between 0.27 and 0.82.

### 3.8. Tissue Histopathology

**Figure 1:**
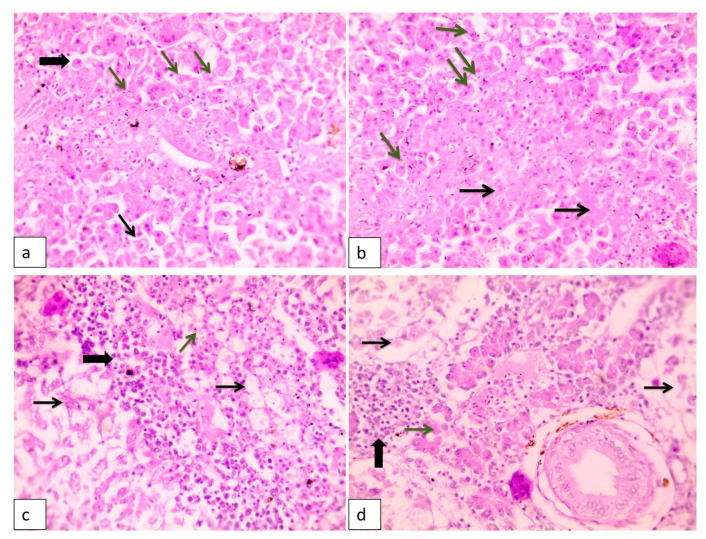
Histopathological lesions in liver of naturally diseased Nile tilapia showing (**a**) hepatocellular dissociation (thin arrow) with many necrotic and apoptotic cells (thick arrow). Few rod-shaped bacteria are inside a hepatocyte cytoplasmic vacuole (green arrows). H&E (hematoxylin and eosin), 400×; (**b**) Widespread hepatocellular necrosis (black arrows) with diffuse, numerous rod-shaped bacteria (green arrows). H&E, 400×; (**c**) Diffuse, severe necrotizing hepatitis. See widespread, severe hepatocellular lysis (arrows) with few bacteria (green arrow) and widespread infiltrations with numerous macrophages and lymphocytes (thick arrow). H&E, 400×; (**d**) Hepatopancreatitis with edema widely separated lytic hepatocytes (arrows) and lysis of hepatopancreas (green arrow) and focal aggregations of inflammatory cells (thick arrow).

**Figure 2:**
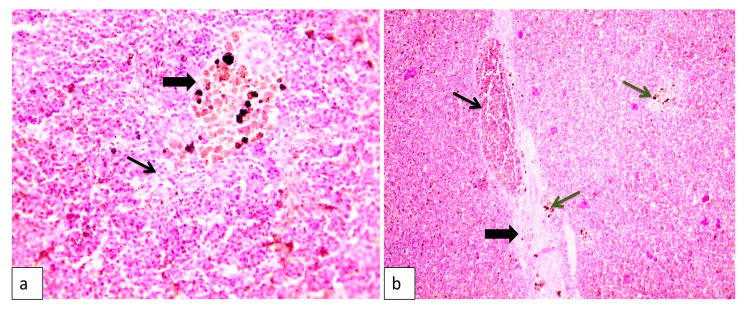
Histopathological lesions in spleen of naturally diseased Nile tilapia showing (**a**) Activation of melanomacrophages (thick arrow) with lymphocytic depletion (arrow), H&E, 400×. (**b**) Congested blood vessels (thin arrow) with edema widely separated splenic cells (thick arrow) and hemosiderosis (green arrows), H&E, 100×.

As shown in Figure 1, the liver of the infected tilapia fish showed widespread hepatocellular necrosis with diffuse, numerous rod-shaped bacteria. Additionally, hepatopancreatitis with edema and widely separated lytic hepatocytes were observed (Figure 1). Lysis of the hepatopancreas and focal aggregations of inflammatory cells were evident as well (Figure 1). The spleen showed an activation of melanomacrophages with lymphocytic depletion. Furthermore, congested blood vessels with edema, widely separated splenic cells, and hemosiderosis were recorded (Figure 2).

## 4. Discussion

The present study provides interesting information relating to summer prevalence, virulence genes, and the antibiogram of motile aeromonads in Nile tilapia fish farms in Egypt. The work also involved water quality assessment in these fish farms together with some histopathological changes that reveal motile aeromonads from fish and water. It is noteworthy to state that water quality is the principal element influencing the aquaculture industry; however, most of the aquaculture sector is carried out as a run-through system with no recirculation of water or effluent treatment prior to its disposal [35]. In the current study, all farms had agriculture drainage canals as water sources except for farm IV, which received additional sewage sources. Such water resources with poor quality from agriculture drainage containing pesticides, and heavy metal pollutants have a potential impact on fish health and disease occurrence. According to the Egyptian legislation (Law No. 124/1983), only irrigation and agriculture drainage water are allowed for fish culturing [6]. Untreated poultry manure was used in all examined farms, which represents a potential health hazard for fish health [36]. As shown in our data, fish mortalities were recorded (200–500 fish/day/pond) in the last two years, especially during summer, which could be attributed to poor water quality and the usage of untreated poultry manure. Poor water quality together with poor management practices might represent a stress factors for cultured fish and thus make them more susceptible to disease outbreaks [37]. However, mortalities dropped to (150–350 fish/day/pond) during the investigation period (March–August 2017) that might be attributed to the use of antibiotics such as tetracyclines and chloramphenicol and the presence of veterinary supervision mainly in farm IV. The physicochemical parameters of water samples from the examined fish farms revealed that some of these evaluated parameters were unsuitable for tilapia culturing according to Egyptian Law standards No. 9/ 2009, reflecting a relatively poor-quality water source in the farms. As observed in our study, most of the measured water quality parameters, particularly at both farms III and IV (Table 2), highly fluctuated from the normal tolerable range for Tilapia as reported in previous studies [38,39]. These observations might be attributed to the usage of agriculture drainage and wastewater as the primary water sources of fish farms III and IV, respectively. As previously mentioned, such types of water are heavily loaded with pollutants such as pesticides, heavy metals, and xenosteroids runoff [40], which worsen the water quality and bioaccumulate in fish tissues. Furthermore, these abiotic stressors affect the overall fish health [41,42], rendering them susceptible to infectious diseases with subsequent economic losses [43].

Bacterial diseases are one of the many challenges facing aquaculture development [44]. Among others, motile *Aeromonas* septicemia (MAS), caused by *Aeromonas* spp., is one of such important bacterial infections, leading to fish mass mortalities and substantial economic losses [17,45,46]. Our results of clinical signs and postmortem lesions on affected fish coincided with previous studies reported elsewhere [47,48,49,50].

In the current results, the bacteriological isolation and biochemical identification of motile aeromonads showed that 80 out of 240 fish samples (33.3%) and one out of eight water samples (12.5%) were positive for motile aeromonads. Our findings are slightly higher to that reported in a previous study in New Zealand where *Aeromonas* were recovered at a rate of 28% [51]; and they are nearly similar to [52], who isolated it at a rate of 26.4% (28/132). As shown in our results, the prevalence of *Aeromonas* was (46.7%, 33.3%, 28.3%, and 25%) from farms IV, III, II, and I, respectively, whereas the highest monthly prevalence rate was recorded during summer (August, June, and July) as (80%, 60%, and 40%, respectively). These findings are related to the poor water quality parameters evaluated in the examined farms, as the highest aeromonads prevalence was noticed in farm IV and farm III, particularly in August and June. These present findings are consistent with other studies reported elsewhere [50,53]. Taken into account, the highest prevalence of *Aeromonas* during summer could be attributed to improper water quality parameters as measured herein, where shifting from normal ranges poses stress on fish with subsequent lowering in their resistance to bacterial infections [50,54].

Antibiotic usage in fish farms is widespread either for the prevention or treatment of bacterial infections in fish [55]; however, their indiscriminate use has led to the emergence of bacterial species with multiple drug resistance all over the world. Our data revealed that the majority of *Aeromonas* isolates showed high resistance rates against the tested antimicrobials, where most of the strains had resistance to at least 4 out of 11 evaluated antibiotics. However, few antibiotics are approved by the Food and Drug Administration (FDA), and the present observations indicate that other non-approved antibiotics tested herein were based on our questionnaire to confirm their illegal usage in some fish farms. This indiscriminate and continued use of antibiotics influences fish health, and it is also considered a public health risk [56]. In line with our results, a previous study reported a high resistance rate of 72% among *Aeromonas* isolates to tetracycline [57]. Furthermore, a higher resistance of up to 100% was described to tetracycline [58]. Conversely, another previous study reported that 100% of *A. hydrophila* isolates showed susceptibility to tetracycline [59]. Our results revealed a high resistance rate among examined isolates up to 75% to trimethoprim–sulphamethoxazole. These findings are higher than those reported in a previous study [60], which recorded 50% resistance of *A. hydrophila* toward this antimicrobial agent. Meanwhile, the same antimicrobial recorded 100% sensitivity of *A. hydrophila* isolates [59]. The resistance rates of our *Aeromonas* isolates to kanamycin reach up to 80%; meanwhile, high sensitivity rates of 28.5% and 87.5% to this antimicrobial were reported elsewhere [60,61]. Interestingly, our *Aeromonas* isolates showed a high resistance rate, up to 80%, to ciprofloxacin, which is contrary to other studies that described ciprofloxacin as the most effective drug against *Aeromonas* infections [58,60,62]. However, a previous study reported 48% resistance of *Aeromonas* isolates to ciprofloxacin [57]. Our study declared that streptomycin, cefotaxime, amoxicillin, and imipenem were the most active drugs against *Aeromonas* isolates. Other studies reported imipenem, chloramphenicol, norfloxacin, and streptomycin as the most highly effective drugs on *Aeromonas* [57,60,63,64].

Studying the antibiogram profiles helps in the forecasting of antibiotic resistance trends. It is obvious that these profiles differ between various studies, which could be accounted for strain-specific traits and selective environmental pressures [65]. Our findings reflect high resistance levels of *Aeromonas* to antimicrobials, which could be attributed to the vast use of antibiotics either as growth promoters or therapeutic agents for various diseases. A MAR index of our isolates showed high ranges between 0.27 and 0.82. In the same context, MAR index ranges of 0.25–0.68 [66] and 0.16–0.42 [50] were recorded in different *Aeromonas* spp. isolated from various aquatic sources.

Virulence genes are the key features in pathogenicity assessments of the microbes, and they can synergistically function [8,67]. Hemolysin and aerolysin are implicated in adhesins, hemagglutinins, and several hydrolytic enzymes that have a crucial role in the pathogenesis of *Aeromonas* spp. [68,69]. As observed in this study, the *aer* gene was detected in a higher number of isolates (it was detected in 42 out of 80), with a percentage of 52.2%. Other virulence genes, including *ahp* and *hyl*, were identified in lower rates as 26.25% and 35%, respectively. Meanwhile, the only *lip* exhibited a very lower detection as 3.75%. Our findings support that this species has a larger matrix of virulence genes in comparison with other species of clinical relevance [70]. Moreover, the existence of more than one virulence factor could share in the pathogenicity of *A. hydrophila* that represents a possible public health risk [71]. Previous studies reported that virulence genes, including *aer* gene, are essential factors in *Aeromonas* spp. [72,73]. The existence of these virulence genes determines the pathogenicity of the infecting microorganisms and seems responsible for specific signs or diseases [72,73]. All *Aeromonas* strains isolated from diseased fish were identified to be virulent, even if they were deficient in one or two virulence genes [74]. Consistently, a previous study demonstrated higher prevalence rates of 81.8%, 88%, and 100% for *aer* gene in isolates of *Aeromonas* spp. [75,76,77]. Furthermore, *Aeromonas* spp. were reported for the presence of *hly* genes (50%) in fish farms in East Delta [76]. Several previous reports revealed that the genes encoding *ahp* and *lip* were identified in *A. hydrophila* isolates and are commonly found in isolates of *Aeromonas* spp. [27]. The *Lip* gene was found in *Aeromonas* isolates at 17.14% [78], while it was highly detected (81.8%) in *A. hydrophila* strains recovered from diseased Nile tilapia [79]. Meanwhile, some previous studies reported that 100% of *A. hydrophila* possess *hyl* and *lip* genes [60,64,80].

In accordance with histopathological changes, as observed in the current study, liver showed different histopathological lesions in the form of widespread hepatocellular necrosis with diffuse, numerous rod-shaped bacteria, hepatopancreatitis, and lysis of the hepatopancreas along with focal aggregations of inflammatory cells. Spleen also showed an activation of melanomacrophages with lymphocytic depletion congested blood vessels. Our findings were consistent with previous studies that showed similar histopathological lesions due to MAS [81,82,83]. These observations were supportive to the motile aeromonads virulence genes herein, as well as the extracellular products, enzymes, and enterotoxins produced by motile aeromonads that together led to a systemic damage to the internal organs, mainly, liver, kidney and spleen with eventual death [9,50,67,81].

## 5. Conclusions

Taken together, the present study provides interesting data with respect to the summer mortality outbreaks in fish from Egypt due to one of the most important etiologies represented by motile aeromonads. Our data also reveal that types of legal and illegal water resources used to fill farms highlighted their crucial role in disease occurrence. Given the above information, these findings could be used as an epidemiological baseline for future references. Our study suggests that further, new strict legislations to use good water for aquaculture must be adopted, along with the monitoring of antibiotic usage and fish farm management.

## Figures and Tables

**Table 1:** The key characteristics of the examined fish farms at Kafrelshiekh province, Egypt.

Key Factors	Farm I	Farm II	Farm III	Farm IV
1- Individual characteristics				
• Age	44	52	51	58
• Gender	Male	Male	Male	Male
• Education	Lower secondary	High education	High education	Upper secondary
2- Farm characteristics				
• Type of production	Semi-intensive	Semi-intensive	Semi-intensive	Semi-intensive
• Size of the farm (m^2^)	12,600	8400	14,700	13,650
• Number of ponds/farms	5	4	5	5
• Fish stocking density/pond	10,000	14,000	18,000	18,500
• Water source	Agriculture drainage	Agriculture drainage	Agriculture drainage	Agriculture + sewage water
• Fish stocking management	Feed supplements, water exchange	Feed supplements, water exchange	Feed supplements, water exchange and night aeration	Feed supplements, water exchange
• Fish production (ton)/feddan	4.5	5	5	3
• Marketing weight-harvest size (g)	300–375	250–300	200–240	300–350
• Use of untreated poultry manure	Yes	Yes	Yes	Yes
• Average previous mortality rate last 2 years/pond/day	200	400	350	500
• Current mortality rate/pond/day	150	300	250	350
• Therapeutic and prophylactic treatment	Humic acid, CaOH powder, probiotics and feed additives	Ca carbonate and manganese	Tetracycline + Probiotics	Chloramphenicol + humic acid and probiotics
• Disinfectants used	QAC	QAC	QAC	Ca hypochlorite
• Veterinary supervision (yes/no)	Occasionally	No	No	Yes

**Table 3:** Physicochemical water parameters of the examined farms at the beginning and at the end of study (March and August 2017).

Parameter	Farm I		Farm II		Farm III		Farm IV		Standard Limits [Reference]
	March	August	March	August	March	August	March	August	
Temp. (°C)	23.5	28.9	21.2	34.1	23.4	30.8	24.7	33.8	28–30 [28]
Ph	7.2	8.6	7.9	8.8	7.5	8.9	8	8.5	6.5–9 [29]
DO (mg/L)	3.4	4		4.5	3.8	4.7	3.1	4	6–14 [30]
EC (mho/cm)	1588	2865	1365	3771	2547	3524	2620	3968	60–2000 µS/cm [31]
TDS (mg/L)	796	1524	674.5	1886	1281.3	1658.4	1342.1	1978.6	≤80 [32]
Ammonia (mg/L)	1.01	1.42	0.55	1.62	1.3	2.6	1.8	3.4	0.6–2.0 [33]
NH_3_–N (mg/L)	0.36	1.17	0.42	1.05	0.49	0.98	0.6	1.23	<0.05–1.0 [29]
Nitrate (mg/L)	0	0	0	0.3	0.2	0.5	0	0.4	<3 [33]
Nitrite (mg/L)	0.1	0.31	0.1	0.34	0.21	0.69	0.07	0.23	<0.5 [34]
Phosphorus (mg/L)	1.17	1.46	0.69	0.92	0.58	0.78	1.87	1.9	0.05–0.5 [31]

DO = dissolved oxygen, EC = electrical conductivity, TDS = total dissolved solids.

**Table 4:** Total prevalence of motile Aeromonads recovered from water and fish samples throughout the study period in the examined farms.

Farms	Water		Fish		Total Examined Samples	
	No. of Positive	%	No. of Positive	%	No. of Positive	%
I	0	0	15	25	15	24.2
II	0	0	20	33.3	20	32.3
III	0	0	17	28.3	17	27.4
IV	1	50	28	46.7	29	46.8
Total	1	12.5	80	33.3	82	33.1

Number of samples; water = 2/farm, fish = 60/farm.

**Table 5:** Monthly prevalence of motile Aeromonads isolated from fish samples in the examined farms.

Months	Farm I	Farm II	Farm III	Farm IV
March	10	0	10	20
April	10	20	30	40
May	20	20	10	40
June	30	50	20	40
July	40	50	60	60
August	40	40	40	80
Total	25	33.3	28.3	46.7

**Table 6:** Molecular characterization of motile Aeromonads isolates and their virulence genes.

Genes	No. of Positive	(%)
Gyrase B (*gyrB*)	65/80	(81.25)
Aerolysin (*aer*)	42/80	(52.5)
Elastase (*ahp*)	21/80	(26.25)
Hemolysin (*hyl*)	28/80	(35)
Lipase (*lip*)	3/80	(3.75)

**Table 7:** Number and percentage of *Aeromonas* isolates resistant to the antibiotics and multiple antibiotic resistances (MAR) index of *Aeromonas* isolates.

Antimicrobial Resistance Pattern		The Multiple Antibiotic Resistance (MAR) Index					
Antibiotics	Number of Resistant *Aeromonas* Isolates (%)	Isolate Number	Antibiotic Resistance Profile *	MAR **	Isolate Number	Antibiotic Resistance Profile *	MAR **
Chloramphenicol (C)	16 (80%)	1	C, CIP, TE, K, SXT, CN, AZM	0.64	35	C, TE, K, SXT, CN, AZM, IPM	0.64
Ciprofloxacin (CIP)	14 (70%)	2	C, CIP, TE, CXT, K, SXT, CN, AZM, S	0.82	36	C, CIP, TE, K, SXT, AZM	0.55
Tetracycline (TE)	13 (65%)	12	C, CIP, TE, K, SXT, AZM, S	0.64	37	CIP, TE, AM, K, SXT, CN	0.55
Amoxicillin (AM)	4 (20%)	13	C, CIP, TE, CXT, K, SXT, CN, AZM, S	0.82	39	C, AM, CXT, K, SXT, CN, AZM	0.64
Cefotaxime (CXT)	6 (30%)	23	C, CIP, TE, K, CN, AZM, S, IPM	0.73	40	C, SXT, CN, S	0.36
Kanamycin (K)	16 (80%)	25	CIP, TE, K, SXT, CN, AZM	0.55	43	C, CIP, K, SXT	0.36
Trimethoprim/sulphamethoxazole (SXT)	17 (75%)	28	C, CIP, TE, CXT, K, SXT, CN, AZM, S	0.82	44	C, CIP, AZM	0.27
Gentamycin (CN)	14 (70%)	29	CIP, AM, CXT, K, SXT, CN, AZM, S	0.73	45	C, TE, K, SXT, AZM	0.45
Azithromycin (AZM)	16 (80%)	33	C, CIP, CXT, K, SXT, CN, AZM, S	0.73	48	C, TE, SXT	0.27
Streptomycin (S)	8 (40%)	34	C, CIP, TE, K, SXT, CN, AZM, S	0.73			
Imipenem (IPM)	2 (10%)						

* Chloramphenicol (C; 30 µg), Ciprofloxacin (CIP; 5 µg), Tetracycline (TE; 5 µg), Amoxicillin (AM; 25 µg), Cefotaxime (CXT; 30 µg), Kanamycin (K; 30 µg), Trimethoprim/sulphamethoxazole (SXT; 25 µg), Gentamycin (CN; 10 µg), Azithromycin (AZM; 15 µg), Streptomycin (S; 10 µg), Imipenem (IPM; 10 µg).** MAR= Multiple antibiotic resistance.

## Supplementary Materials

The following are available online at https://www.mdpi.com/2076-2615/10/8/1432/s1, Figure S1: Fish mortalities on water surface and on the pond banks. Figure S2: Clinical signs of freshly dead *O. niloticus* fish; hemorrhages on the skin, fins and vent opening, loosened scales with deep skin ulceration and fin erosions, distended abdomen and exophthalmia. Figure S3: Agarose gel electrophoresis of *Aeromonas* isolates from different sources using 100-bp molecular standard size ladder. Table S1: The number, ranges of lengths, and weights of collected tilapia fish during the study period (March–August 2017). Table S2: Phenotypic characterization results of Aeromonas species retrieved from water and fish samples in the examined fish farms.
