# Supplementary material for: Investigation of the Prevalence, Virulence Genes, and Antibiogram of Motile Aeromonads Isolated from Nile Tilapia Fish Farms in Egypt and Assessment of their Water Quality
Source: Animals (Basel). 2020 Aug 16;10(8):1432. doi: 10.3390/ani10081432 (PMC7459692; doi:10.3390/ani10081432)
Supplement: Supplementary file 1 [file animals-10-01432-s001.zip › animals-880829-supplementary.docx]

**Supplementary tables:**

**Table S1.** The number, ranges of lengths, and weights of collected tilapia fish during the study period (March – August 2017)

| **Farms** | **Number of collected fish (#)** | | **Range of Length (cm)** | **Range of weight (g)** |
| --- | --- | --- | --- | --- |
|  | **Healthy** | **Diseased** |  |  |
|  |  | |  |  |
| **Farm I** | 20 | 40 | 7.85 – 16.5 | 16.35 – 186.57 |
| **Farm II** | 15 | 45 | 8.48 – 16.71 | 18.79 – 196.85 |
|  |  | |  |  |
| **Farm III** | 25 | 35 | 7.75 – 13.79 | 30.04 – 163.54 |
| **Farm IV** | 30 | 30 | 9.24 –15.63 | 18.80 – 181.69 |

**Table S2.** Phenotypic characterization results of *Aeromonas species* retrieved from water and fish samples in the examined fish farms

| Phenotypic test | Test result | Positive *Aeromonas hydrophila* isolates % | | |
| --- | --- | --- | --- | --- |
|  |  | **Water (n=8)** | | **Fish (n=240)** |
| Aeromonas selective agar base medium | Small dark green convex bull eye shaped colonies with dark green center | 37.5 | 83.3 | |
| Gram staining | - | 37.5 | 79.2 | |
| Motility | + | 37.5 | 62.5 | |
| Catalase | + | 25 | 50 | |
| Oxidase | + | 25 | 50 | |
| Aesculin hydrolysis | + | 25 | 33.3 | |
| Acid/gas from glucose | +/+ | 12.5 | 33.3 | |

**Supplementary figures:**


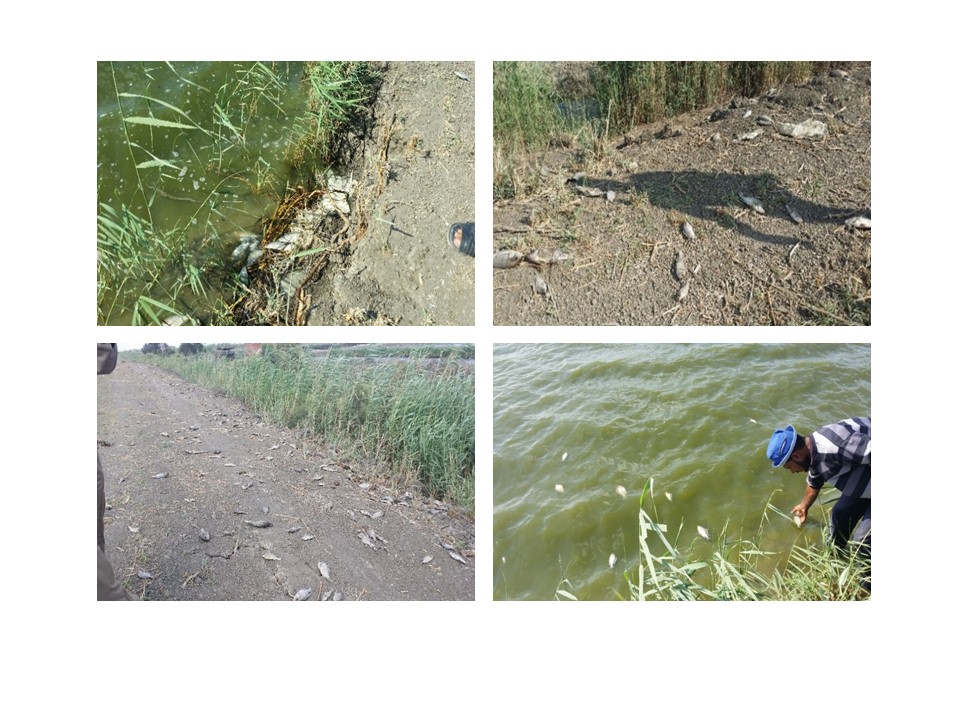


**Fig.S1.** Fish mortalities on water surface and on the pond banks.


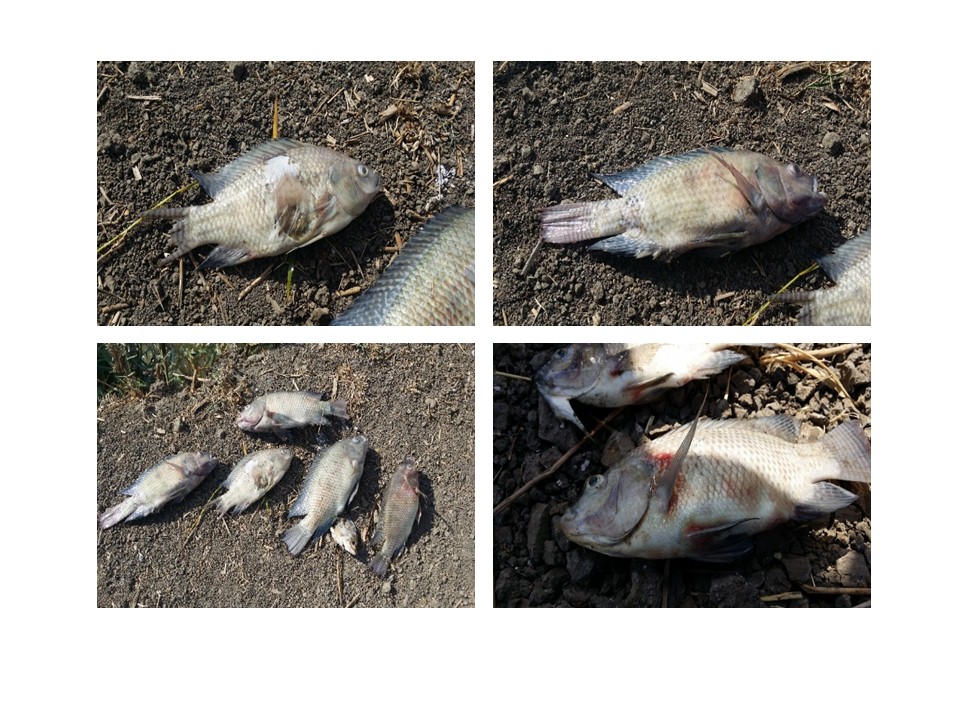


**Figure S2.** Clinical signs of freshly dead *O. niloticus* fish; hemorrhages on the skin, fins & vent opening, loosened scales with deep skin ulceration and fin erosions, distended abdomen and exophthalmia.


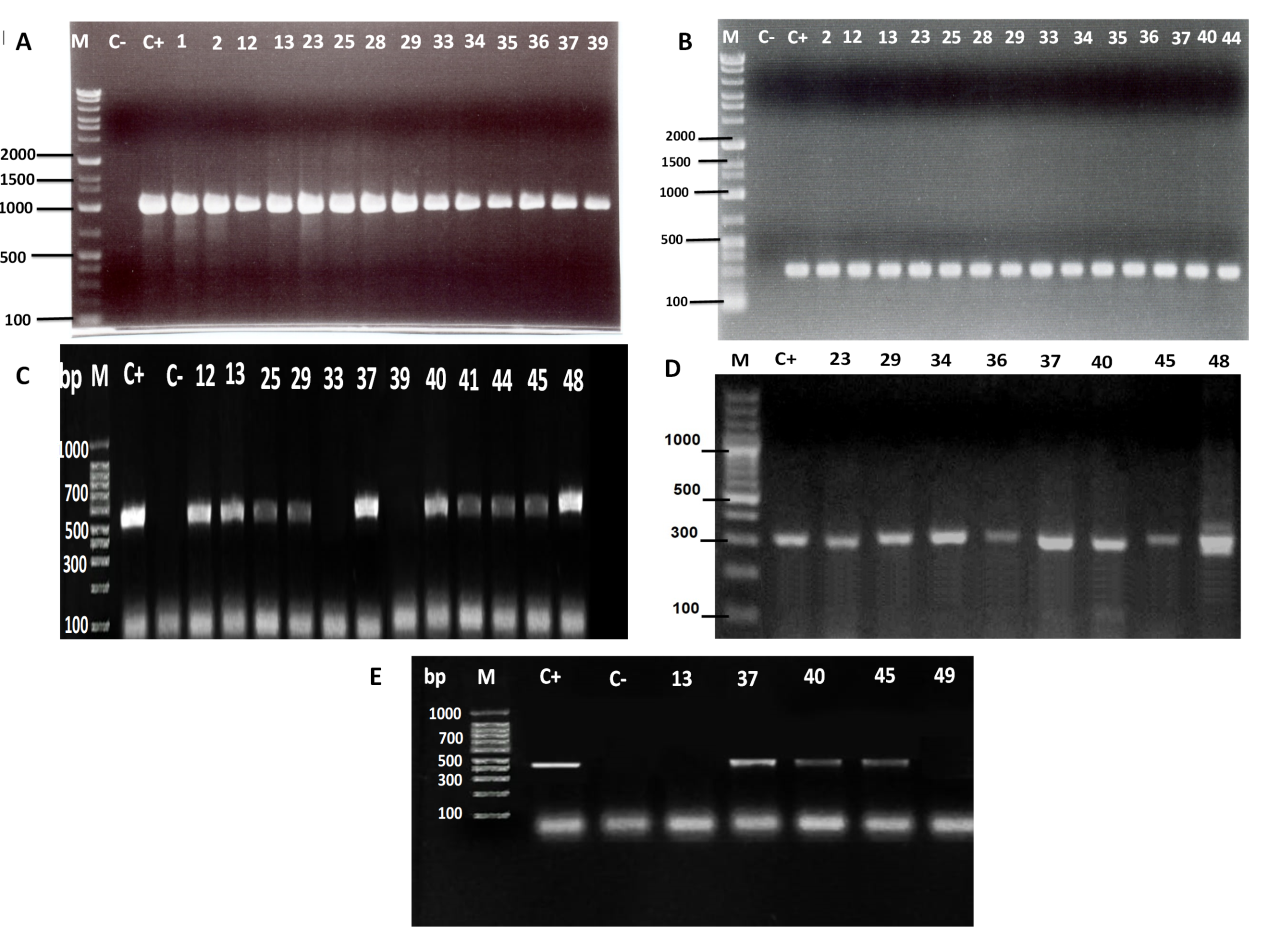


**Figure S3.** Agarose gel electrophoresis of *Aeromonas* isolates from different sources using 100-bp molecular standard size ladder for: A) gyr-B gene amplification with amplicon size of 1100 bp, Lane1, Lane 2, negative control (no template); Lane 3, *A. hydrophila* positive control; Lane 4-17 fish isolates; B) aerA gene amplification with amplicon size of 301 bp, Lane1, Lane 2, negative control (no template); Lane 3, *A. hydrophila* positive control; Lane 4-17 fish isolates; C) ahp gene amplification with amplicon size of 540 bp, Lane1, Lane 2, *A. hydrophila* positive control; Lane 3, negative control (no template); Lane 4-14 fish isolates; D) hemolysin gene amplification of *Aeromonas* isolates from different sources with amplicon size of 326 bp. Lane 2, *A. hydrophila* positive control; Lane 3-10 fish isolates; E) lipase gene amplification with amplicon size of 383-389 bp. Lane 2, *A. hydrophila* positive control, Lane 3, negative control (no template); Lane 4-8 fish isolates.
